# The complete mitochondrial genome of *Eutrichosiphum pasaniae* (Okajima, 1908) (Hemiptera: Aphididae: Greenideinae)

**DOI:** 10.1080/23802359.2020.1831997

**Published:** 2020-11-03

**Authors:** Cailing Li, Liyun Jiang, Xiaolu Zhang, Jing Chen, Gexia Qiao

**Affiliations:** a Key Laboratory of Zoological Systematics and Evolution, Institute of Zoology, Chinese Academy of Sciences, Beijing, China; b Institutes of Physical Science and Information Technology, Anhui University, Hefei, China; c College of Life Sciences, University of Chinese Academy of Sciences, Beijing, China

**Keywords:** Mitogenome, aphid, repeat region, phylogeny

## Abstract

In this study, we sequenced the complete mitochondrial genome of *Eutrichosiphum pasaniae* through Illumina platform. The circular mitogenome is 16,500 bp in length and composed of 13 protein-coding genes (PCGs), 22 transfer RNA genes (tRNAs), 2 ribosomal RNA genes (rRNAs), a large control region and a special repeat region. The nucleotide composition of whole mitogenome is strongly AT-biased (85.5%). All PCGs start with ATN and end with TAA except for *cox1* which terminates with an incomplete stop codon T. All tRNAs have a typical clover-leaf secondary structure except for *trnS (AGN)*. The lengths of *rrnL*, *rrnS* and control region are 1276, 774 and 996 bp, respectively. The repeat region with a length of 909 bp is located between *trnE* and *trnF* and consists of 4.1 repeat units. The phylogenetic tree supports the sister relationship of *Eutrichosiphum pasaniae* and *Greenidea psidii*.

The aphid species *Eutrichosiphum pasaniae* (Okajima, 1908) (Hemiptera: Aphididae: Greenideinae) distributes in eastern and southeastern Asia and mainly feeds on leaves and young shoots of the plants of *Lithocarpus* and *Castanopsis* (Blackman and Eastop 2020). In the present study, we sequenced the complete mitochondrial genome of *E. pasaniae* through the Illumina platform. The *E. pasaniae* samples were collected from *Castanopsis uraiana* in Daren Township, Taiwan, China (22.3750°N, 120.8592°E) and deposited in the National Zoological Museum of China, Institute of Zoology, Chinese Academy of Science, Beijing, China (NZMC no. 39256).

The circular mitochondrial genome of *E. pasaniae* is 16,500 bp long (GenBank accession number MT883997) and includes 13 protein-coding genes (PCGs), 22 transfer RNA genes (tRNAs), 2 ribosomal RNA genes (rRNAs), a control region and a special repeat region between *trnE* and *trnF*. The gene order is identical to the inferred ancestral arrangement of insects (Clary and Wolstenholme 1985). The majority strand contains 9 PCGs and 14 tRNAs, while the remaining genes are located on the minority strand. The overall nucleotide composition of *E. pasaniae* mitogenome is 46.6% A, 38.9% T, 5.5% G and 9.1% C, which is strongly AT-biased (85.5%). In the whole mitogenome, there are 21 intergenic spacers ranging from 1 to 51 bp and 8 gene overlapping regions ranging from 1 to 20 bp.

Thirteen PCGs are initiated by the standard ATN and ended with TAA expect for *cox1*, which uses an incomplete stop codon T. The tRNA genes range from 62 to 73 bp in length and are predicted to possess a classical clover-leaf secondary structure expect for *trnS (AGN)*, the dihydrouridine (DHU) arm of which forms a simple loop. The lengths of *rrnL* and *rrnS* genes are 1276 and 774 bp, with an A + T content of 85.3 and 83.8%, respectively. The control region is 996 bp long and located between *rrnS* and *trnI*, with an A + T content of 92.2%. A large repeat region is present between *trnE* and *trnF*, which is unique and species-specific in aphids (Wang et al. 2015). The repeat region is 909 bp long with an A + T content of 87.6% and consists of a 222-bp repeat unit which is repeated 4.1 times.

To perform the phylogenetic analysis, we used the whole mitogenome sequences of *E. pasaniae* and 24 other aphid species. The maximum-likelihood phylogenetic tree was constructed with RAxML v8.2.10 (Stamatakis 2014). The subfamily Greenideinae was monophyletic and *E. pasaniae* was placed as a sister to *Greenidea psidii* (Figure 1).

**Figure 1.**
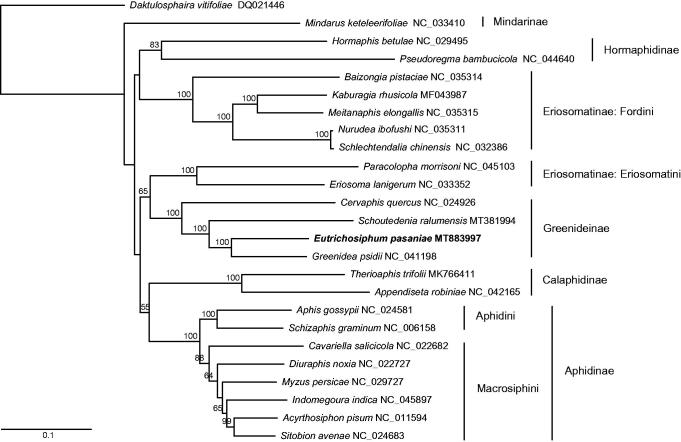
The maximum-likelihood tree of *Eutrichosiphum pasaniae* and 24 other aphid species based on whole mitogenomes. Numbers above the branches indicate bootstrap values (>50%).

## Data Availability

The data that support the findings of this study are openly available in Dryad at https://doi.org/10.5061/dryad.zkh189388.
